# Supplementary material for: A Global Screen for Assembly State Changes of the Mitotic Proteome by SEC-SWATH-MS
Source: Cell Syst. 2020 Feb 26;10(2):133–155.e6. doi: 10.1016/j.cels.2020.01.001 (PMC7042714; doi:10.1016/j.cels.2020.01.001)

Q9BY32 | ITPA_HUMAN | ITPA C20orf37 My049 OK/SW−cl.9
Monomer MW [kDa]: 21.446 Monomer expected elution fraction: 54

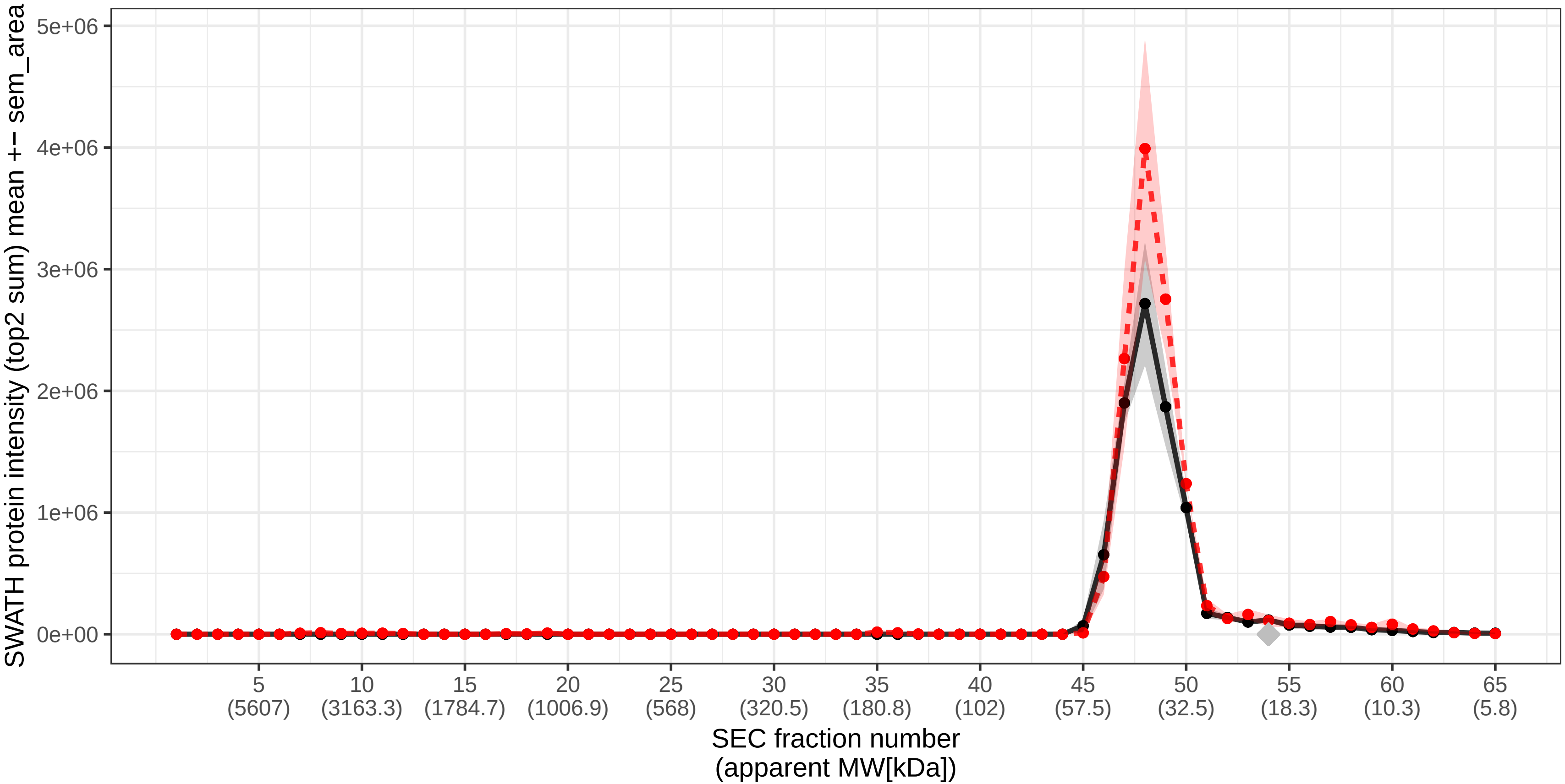

Supplement: Data S1. SEC-SWATH-MS Protein Chromatograms, Related to Figure 1 [file mmc6.zip › SECchrom_Q9BY32_ITPA_HUMAN_ITPA_C20orf37_My049_OK_SW-cl.9.pdf]
